# Suppressing epileptic activity in a neural mass model using a closed-loop proportional-integral controller

**DOI:** 10.1038/srep27344

**Published:** 2016-06-07

**Authors:** Junsong Wang, Ernst Niebur, Jinyu Hu, Xiaoli Li

**Affiliations:** 1 School of Biomedical Engineering, Tianjin Medical University, Tianjin 300070, China; 2 Zanvyl Krieger Mind/Brain Institute and Solomon Snyder Department of Neuroscience, Johns Hopkins University, Baltimore, MD 21218, USA; 3 Division of Immunology and Rheumatology, Department of Medicine, Stanford University, Stanford, CA 94305, USA; 4 National Key Laboratory of Cognitive Neuroscience and Learning, Beijing Normal University, Beijing 100875, China

## Abstract

Closed-loop control is a promising deep brain stimulation (DBS) strategy that could be used to suppress high-amplitude epileptic activity. However, there are currently no analytical approaches to determine the stimulation parameters for effective and safe treatment protocols. Proportional-integral (PI) control is the most extensively used closed-loop control scheme in the field of control engineering because of its simple implementation and perfect performance. In this study, we took Jansen’s neural mass model (NMM) as a test bed to develop a PI-type closed-loop controller for suppressing epileptic activity. A graphical stability analysis method was employed to determine the stabilizing region of the PI controller in the control parameter space, which provided a theoretical guideline for the choice of the PI control parameters. Furthermore, we established the relationship between the parameters of the PI controller and the parameters of the NMM in the form of a stabilizing region, which provided insights into the mechanisms that may suppress epileptic activity in the NMM. The simulation results demonstrated the validity and effectiveness of the proposed closed-loop PI control scheme.

Epilepsy is thought to be a dynamical disorder of the brain at the systems level^1^,^2^,^3^, which makes it particularly suitable to be studied from the perspectives of computational modelling and system theory^1^,^4^,^5^. Closed-loop control is a promising deep brain stimulation (DBS) strategy for suppressing abnormal neural activities, such as epilepsy and Parkinson’s disease, and it has thus become the focus of current experimental and theoretical studies^6^,^7^,^8^,^9^,^10^,^11^,^12^,^13^,^14^,^15^,^16^,^17^,^18^.

Control theory is an emerging method that is used to develop closed-loop controllers for suppressing epileptic seizures^11^, and some advanced control strategies are involved in the design of closed-loop DBS control^10^,^13^,^14^. A closed-loop controller was developed based on a recursively identified autoregressive model that described the relationship between stimulation input and local field potential output^10^. A responsive neuron modulator, based on a radial basis function neural network, was employed to control seizure-like events in a computational model of epilepsy^13^. In addition, optimal control theory was used to design a desynchronizing control stimulus for a network of pathologically synchronized neurons^14^. Although these control schemes performed excellently, the control algorithms were somewhat complex. These results suggest that a simple control algorithm may be preferred in closed-loop control of epileptic activity.

A proportional-integral-derivative (PID) controller is the most extensively used closed-loop controller in the field of control engineering because of its simple implementation and robust performance^19^. In this context, the PID-type controller was introduced to control various pathological neural activities^12^,^20^,^21^,^22^,^23^. Proportional and proportional-derivative amplitude control were proposed for the closed-loop DBS of patients with Parkinson’s disease^24^. Proportional feedback stimulation was employed to control seizures in rats^25^. Integral feedback control was developed for the charge-balanced suppression of epileptic seizures^26^ and the modulation of brain rhythms in Parkinson’s disease^27^. Proportional controllers, differential controllers and filter controllers were used to eliminate seizing activity in a mathematical model of human cortical, electrical activity^28^. A reactive and adaptive control scheme was proposed to suppress epileptiform discharges in realistic, computational, neuronal models with bistability^29^. In this model, the cumulative sum of the difference between the measured ictality and the pre-defined acceptable limit over all previous epochs was used to determine the modulating factor for the instantaneous stimulation strength during the current epoch. Therefore, the control scheme was, in essence, integral feedback control in a discrete form. Furthermore, delayed feedback control is another representative control scheme that has been used to suppress pathological brain rhythms by desynchronizing the neural activities^30^,^31^.

PID-type controllers have achieved great success in controlling various pathological neural activities. However, the control parameters are currently chosen empirically by using a “trial and error” approach. This makes the outcome strongly dependent on the designer’s experience. Additionally, the work is time-consuming and its efficiency is not guaranteed, which makes the choice of control parameters quite challenging. Thus, an analytical design method of the PID-type controller is urgently needed. Furthermore, because epilepsy is caused by an unbalance between excitation and inhibition^32^, the control parameters should be related to the excitatory and inhibitory parameters. However, it still remains unclear how to build a quantitative relationship between the two sides.

Computational models have become attractive due to their ability to model complex neurological phenomena with relative ease^1^,^33^,^34^,^35^. A neural mass model is based on a biologically plausible parameterization of the layered neocortex dynamic behaviour. Neural mass model was initially proposed to study the origin of the alpha rhythm, and subsequently improved and extended to describe more general cortical, electrical activities^34^, such as electroencephalogram (EEG)^36^,^37^, functional magnetic resonance imaging (FMRI) signals^38^ and event-related potentials^39^. Specifically, the NMM was successfully used to generate epileptic activity similar to that experimentally observed^40^,^41^,^42^,^43^. Jansen’s neural mass model (NMM) is characterized by the interaction of the interlinked excitatory and inhibitory feedback loops^44^. Previous studies have demonstrated that Jansen’s NMM can generate high-amplitude epileptic activity caused by the abnormal values of the external input^43^,^45^ and the connection strength of the model^41^. Our recent bifurcation study found that the imbalance of the excitatory and inhibitory feedback loops in Jansen’s NMM resulted in the generation of high-amplitude epileptic activity^42^. Therefore, we used Jansen’s NMM as a test bed to develop the closed-loop PI controller for suppressing epileptic activity in the model (for simplicity, we use NMM to represent Jansen’s NMM in the remainder of this article).

Here, we used feedback control theory to develop a proportional-integral (PI) controller to suppress the high-amplitude epileptic activity in the NMM. A graphical stability analysis method was employed to determine the stabilizing region of the PI controller in the control parameter space. Our main goal was to develop an analytical design method for a PI controller that suppresses epileptic activity and to provide theoretical guidelines for choosing PI control parameters. If the PI controller stabilizing region was known for a given set of excitatory and inhibitory parameters of the NMM, then the time-consuming stability check for each set of control parameters could be avoided and the PI controller tuning time could be reduced. Furthermore, we determined the relationship between the parameters of the PI controller and the excitatory and inhibitory parameters of the NMM in the form of a stabilizing region, which was helpful for understanding the mechanism of suppressing epileptic activity.

## Methods

### Model

**Figure 1:**
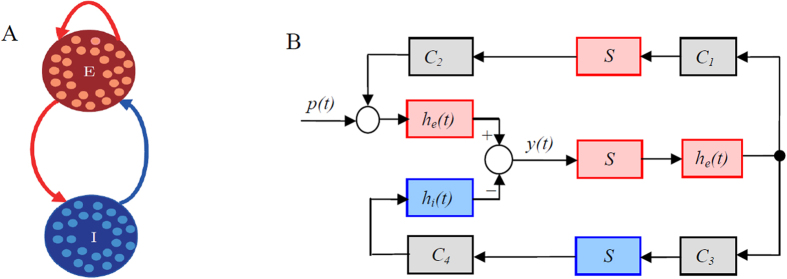
Structure of the neural mass model. (**A**) The schematic diagram of the NMM. “E” and “I” represent excitatory and inhibitory subpopulations, respectively, that are defined as groups of statistically similar excitatory or inhibitory neurons that share the same inputs and connectivity. (**B**) Block diagram of the NMM. The red and blue blocks correspond to excitatory and inhibitory subpopulations, respectively.

The schematic and block diagram of the NMM is shown in Fig. 1A,B^44^, respectively. The NMM was composed of the following three interacting subpopulations: the main subpopulation (middle part), the excitatory (top part) and inhibitory (bottom part) feedback subpopulations. *C*_1_, *C*_2_, *C*_3_ and *C*_4_ are connectivity constants that represent interactions between the subpopulations and characterize the average numbers of synaptic contacts; *p*(*t*) is the input of the NMM and modelled by Gaussian noise; and *y*(*t*) is the output of the NMM, corresponding to the average synaptic activity of the pyramidal cells, that is interpreted as an EEG signal.

Each subpopulation of the NMM was composed of an excitatory, *h*_*e*_(*t*), or inhibitory synaptic dynamic function, *h*_*i*_(*t*), and a sigmoid static function, *S*(*v*). The synaptic functions, *h*_*e*_(*t*) and *h*_*i*_(*t*), transform the average pre-synaptic firing rates into average post-synaptic membrane potentials and are defined as 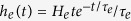 and 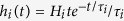
44, where *H*_*e*_ and *H*_*i*_ represent the excitatory and inhibitory synaptic strengths and *τ*_*e*_ and *τ*_*i*_ are the membrane time constants. The sigmoid function, *S*(*v*), transforms the average membrane potential into the average firing rate and is defined as 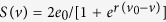
46, where 2*e*_0_ is the maximum firing rate, *v*_0_ is the post-synaptic potential corresponding to a firing rate of *e*_0_, and *r* describes the steepness of the sigmoid function. The parameter values of the NMM are listed in Table 1^44^.

### Graphical stability analysis method

A graphical stability analysis method was employed to determine the stabilizing region of PI controller for suppressing epileptic activity in the NMM. The key point of designing the PI controller was to choose the control parameter values such that homeostasis of the NMM was maintained. To achieve this objective, we wanted to determine the stabilizing region of the PI controller.

If it is assumed that the characteristic polynomial of one control system is denoted as Δ(*s*), then all of the coefficients of Δ(*s*) are real; therefore, the characteristic roots of Δ(*s*) must be complex conjugates. According to linear stability theory^47^, the stability boundary of the control system is defined by Δ(*jω*) = 0. Hence, it is sufficient to consider the following two cases: *ω* = 0 and *ω* ∈ (0, ∞). The stability boundary of the control system was defined as follows^48^:


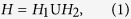


where


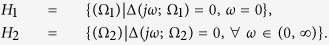


where Ω_1_ and Ω_2_ represent the control parameter sets of the employed controller to be determined for the two cases *ω* = 0 and *ω* ∈ (0, ∞), respectively. In this study, Equation (1) was utilized to determine the stabilizing region of the PI controller in the control parameters space.

## Results and Discussion

### Control Scheme

The control scheme was developed based on the fact that epileptic activity can be characterized as high-amplitude limit cycle oscillation born in Hopf bifurcation^4^,^28^,^31^,^33^,^36^,^41^,^42^,^49^,^50^,^51^,^52^,^53^, which indicates that the fixed point of the NMM lost its stability. Thus, we aimed to design a PI controller to stabilize the unstable, fixed point to prevent the generation of the Hopf bifurcation and to further suppress high-amplitude epileptic activity.

Epilepsy is thought to be caused by an imbalance between excitation and inhibition resulted from hyper-excitation or low inhibition^32^. Thus, the design objective was to determine the PI controller parameter values that stabilized the unstable NMM caused by abnormally large excitatory or small inhibitory parameter values^32^,^42^ such that homeostasis of the NMM was maintained and the high-amplitude epileptic activity was suppressed. To do that, we employed a graphical stability analysis method that determined the stabilizing region of the PI controller in the parameter space.

**Figure 2:**
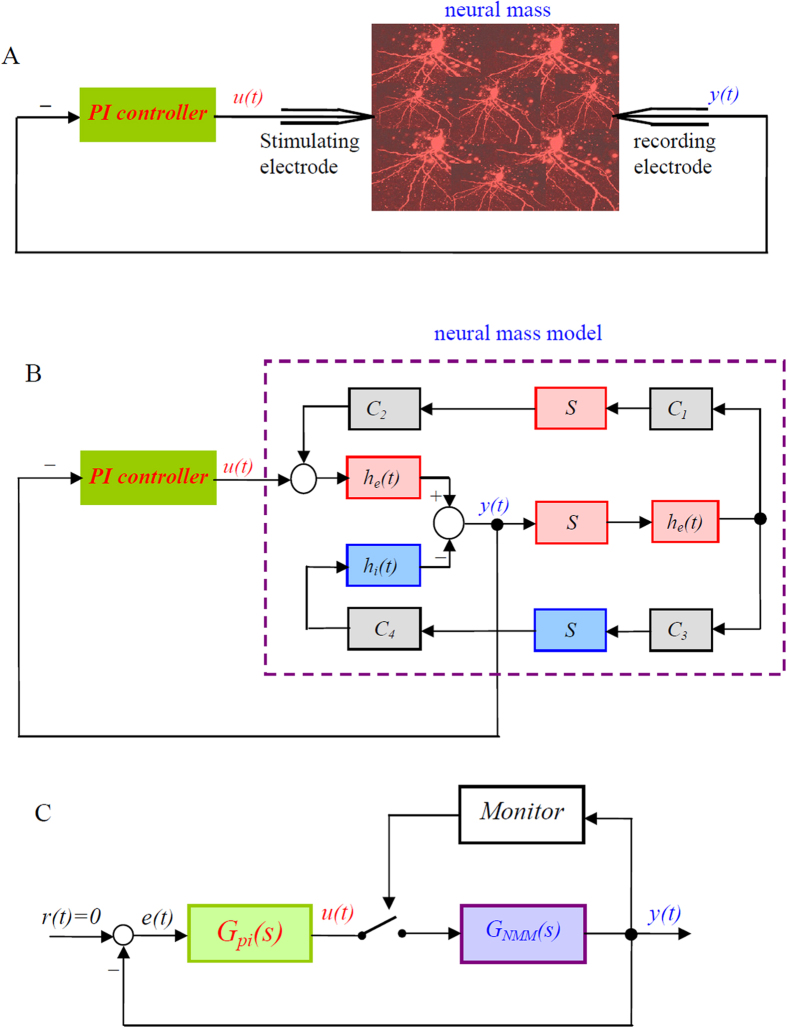
The proposed PI control scheme for suppressing epileptic activity in the NMM. (**A**) Schematic diagram of the PI-based control scheme. (**B**) Block diagram of the PI-based control scheme that modelled the activities of the neural mass using the NMM. The purple dashed block represents the NMM. (**C**) The simplified equivalent form of the proposed PI control scheme that replaced the purple dashed block in Fig. 2B with the transfer function *G*_*NMM*_(*s*) of the NMM.

The proposed PI control scheme for suppressing epileptic activity is shown in Fig. 2A, where *u*(*t*) is the output of PI controller, corresponding to the electrical stimulation signals, and *y*(*t*) represents the local field potential of a neural mass. The local electrical field in the neural mass was measured by the recording electrode and feeds back to the PI controller via the stimulating electrode. By using the NMM to model activity of the neural mass in Fig. 2A, we derived the block diagram of the proposed control scheme, shown in Fig. 2B, where *y*(*t*) is the output of the NMM and *u*(*t*) is the output of the PI controller.

To take advantage of the graphical stability analysis method used to design the PI controller, we replaced the nonlinear sigmoid function, *S*(*v*), with its linear approximation around the equilibrium point, *v* = *v*_0_, as follows: 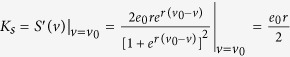, where *S*′(*v*) represents the derivative of *S*(*v*).

According to Fig. 2B, we derived the transfer function of the NMM as follows:


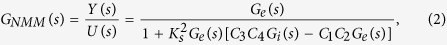


where *Y*(*s*) and *U*(*s*) are the Laplace transform of *y*(*t*) and *u*(*t*), respectively. 
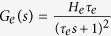
 and 
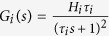
 are the Laplace transform of *h*_*e*_(*t*) and *h*_*i*_(*t*) in the NMM, respectively.

Figure 2B can be simplified as a standard form of typical control systems, shown in Fig. 2C, where *r*(*t*) is the desired output of the NMM, *e*(*t*) is the error signal of the closed-loop control system and the system is defined as *e*(*t*) = *r*(*t*) − *y*(*t*). For simplicity, in the remainder of the article, we will use PI-NMM to represent the closed-loop control system composed of the PI controller and the NMM.

In addition to epileptic activity, the NMM may feature multiple parallel states, such as low-amplitude oscillation and fluctuation output at a fixed point^33^,^50^,^51^,^54^,^55^. Thus, as shown in Fig. 2C, the feedback loop was closed only when the amplitude of the output of the NMM exceeded a predetermined threshold. Correspondingly, the control signal *u*(*t*) is defined as


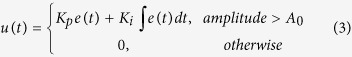


where *K*_*p*_ and *K*_*i*_ are the proportional and integral coefficients of the PI controller, respectively. *A*_0_ is the predetermined amplitude threshold beyond which stimulation is applied.

By conducting a Laplace transform of Equation (3), we can obtain the transfer function of the PI controller as follows:


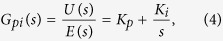


where *E*(*s*) is the Laplace transform of the error signal, *e*(*t*).

According to feedback control principles^47^, the control objective of a feedback control system is to make the error signal *e*(*t*) → 0. This indicates that the output of the PI-NMM control system will approach the desired output under the control of the PI controller, i.e., *y*(*t*) → *r*(*t*). In this study, our goal in implementing the PI controller was to suppress the high-amplitude epileptic activity in the NMM; thus, the desired output of the PI-NMM control system was set to zero, i.e., *r*(*t*) = 0.

### Theoretical results of the stability analysis

To determine the stabilizing region of the PI controller in the (*K*_*p*_, *K*_*i*_) parameter space in which the PI-NMM control system was stable, we used the graphical stability analysis method to determine the stability boundary of the PI controller in the *K*_*p*_, *K*_*i*_ plane.

According to Fig. 2C, we can derive the characteristic equation of the PI-NMM control system as


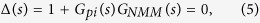


where Δ(*s*) is the characteristic polynomial of the PI-NMM control system.

Defining *s* = *jω* and substituting it into Equation (5), one can obtain


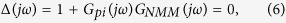


Equation (6) defines the stability boundary of the PI-NMM control system.

According to the graphical stability analysis method shown in Equation (1), we can discuss the two cases *ω* = 0 and *ω* > 0.

*Case 1: ω* = 0. Substituting Equations (2) and (4) into Equation (5), we can derive the characteristic equation of the PI-NMM control system as follows


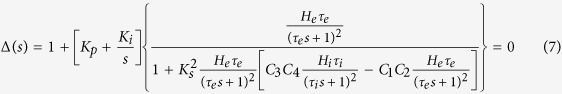


Let *s* = *jω*. Thus, Equation (7) can be rewritten as


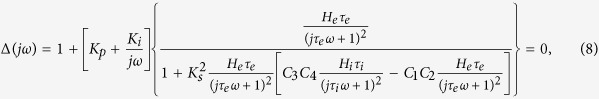


For *ω* = 0, Equation (8) can be simplified as


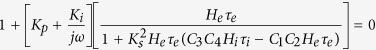


We can derive


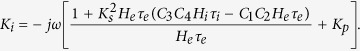


Thus, we can obtain the stability boundary for the case *ω* = 0, i.e., *H*_1_, as follows:


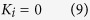


**Figure 3:**
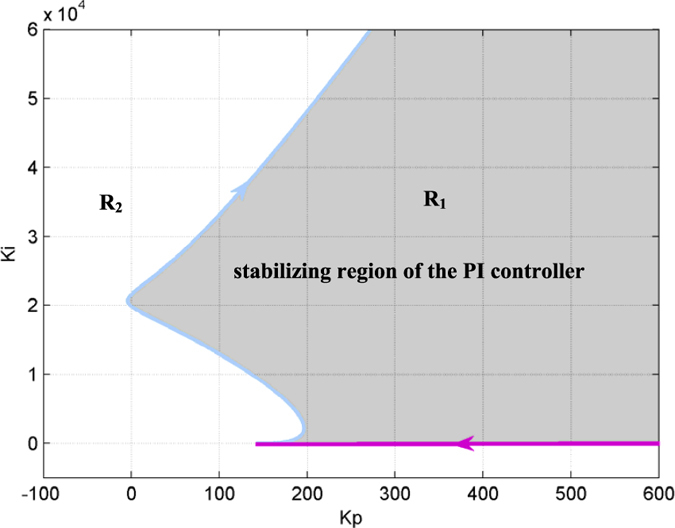
The stabilizing region of the PI controller to suppress epileptic activity in the NMM (*H*_*e*_ = 4.5 mV). The arrows represent the direction of the curve (*K*_*p*_(*ω*), *K*_*i*_(*ω*)) in which *ω* increases. The light blue curve is defined by Equation (13), and the purple line is defined by Equation (9). The parameter space to the right of the curve and above the line is the stabilizing region of the PI controller.

This equation is a line in the (*K*_*p*_, *K*_*i*_) plane that is shown in Fig. 3 (the purple line).

Next, according to Equation (7), we can further derive the characteristic equation of the PI-NMM control system as follows:


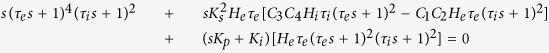


Note that the coefficient of *s*^7^ in the characteristic polynomial, i.e., 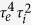, is positive. According to the Routh stability criterion^47^, if the system is stable, then all of the coefficients of the characteristic equation should have the same sign. The constant term should also be positive, i.e., *K*_*i*_*H*_*e*_*τ*_*e*_ > 0. Thus, it follows that


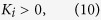


Therefore, we can obtain the following lemma 1.

Lemma 1 For the case *ω* = 0, the region above the *K*_*i*_ axis in the (*K*_*p*_, *K*_*i*_) plane is the stabilizing region of the PI controller.

*Case 2: ω* = 0. Supposing that *δ*_*R_NMM*_(*ω*) and *δ*_*I_NMM*_(*ω*) are the real and the imaginary components of *G*_*NMM*_(*jω*), respectively, then Equation (6) can be rewritten as


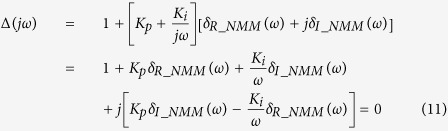


Equation (11) can be split into the following two parts


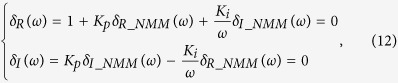


where *δ*_*R*_(*ω*) and *δ*_*I*_(*ω*) are the real and imaginary components of Δ(*jω*), respectively.

Note that both *δ*_*R*_(*ω*) and *δ*_*I*_(*ω*) are dependent on *K*_*p*_ and *K*_*i*_. Thus, the stability of the characteristic Equation (12) can be investigated in the parameter space (*K*_*p*_, *K*_*i*_).

According to Equation (12), we can further derive


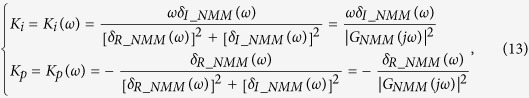


where 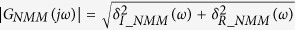 is the complex modulus of *G*_*NMM*_(*jω*).

Equation (13) defines the stabilizing region *H*_2_ of the PI controller in the (*K*_*p*_, *K*_*i*_) plane for the case *ω* > 0. According to Equation (13), we can draw the stability boundary curve (*K*_*p*_(*ω*), *K*_*i*_(*ω*)) for *ω* > 0, as shown in Fig. 3 (the light blue curve).

According to Equation (1), the stability boundary is composed of the stability boundary line defined by Equation (9) and the stability boundary curve defined by Equation (13). As illustrated in Fig. 3, the stability line and the stability boundary curve divide the (*K*_*p*_, *K*_*i*_) plane into two regions, which are denoted *R*_1_ and *R*_2_, respectively. In the following section, we investigate which is the stabilizing region of the PI controller.

Next, we introduce the following proposition^56^. If one travelled along the curve defined by *H*_2_ in the direction of increasing *ω*, then the right side is the stabilizing parameter region where det *J* < 0 and the left is where det *J* > 0. Here, *J* is the Jacobian matrix defined as


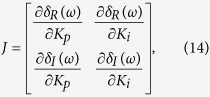


From Equation (12), we obtain 
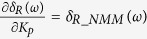
, 
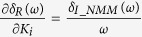
, 
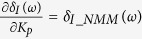
 and 
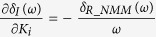
.

Substituting the above four equations into Equation (14), we can obtain 
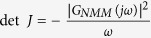
. Because *ω* > 0 and 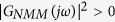, it follows that


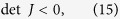


Therefore, we derive the following lemma 2.

Lemma 2 For the case *ω* > 0, following the curve (*K*_*p*_(*ω*), *K*(*ω*)) in the direction of increasing *ω*, the parameter space to the right of the curve defined by Equation (13) is the stabilizing region of the PI controller.

### Main theoretical results

Combining Lemma 1 with Lemma 2 yields the following theorem.

Theorem 1 For the PI-NMM control system depicted in Fig. 2, the exact stabilizing parameter region of the PI controller is the parameter space above the line defined by Equation (9) and to the right of the curve defined by Equation (13) if it is followed in the direction of increasing *ω*.

Thus, according to Theorem 1, the region denoted by *R*_1_ in Fig. 3 was identified as the stabilizing region of parameters (*K*_*p*_, *K*_*i*_), and *R*_2_ was the destabilizing parameter region.

### Effect of excitatory and inhibitory NMM parameters on the stabilizing region of the PI controller

Epileptic activity is caused by the imbalance of excitation and inhibition in the NMM, which is caused by extremely large excitatory, *H*_*e*_, or small inhibitory parameters, *H*_*i*_, respectively. Therefore, we discussed the effect of the two parameters on the stabilizing region of the PI controller.

**Figure 4:**
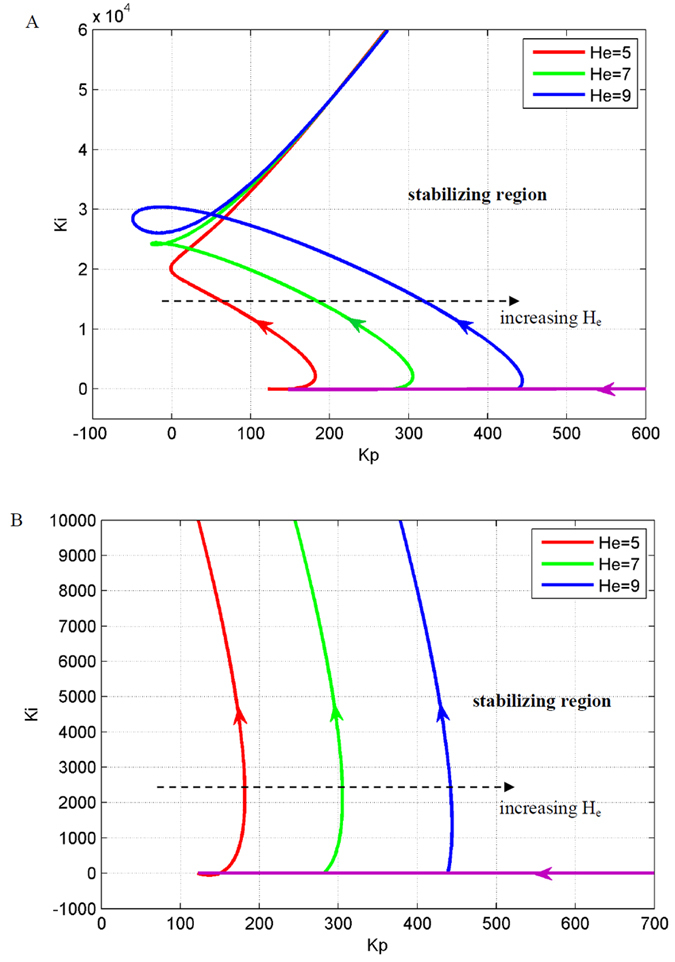
The effect of excitatory parameter, *H*_*e*_, on the stabilizing region of the PI controller, (**B**) is an enlarged version of (**A**). The parameter space to the right of the red, green and blue curves are the stabilizing region of the PI controller for suppressing epileptic activity in the NMM with *H*_*e*_ = 5, 7 and 9, respectively.

**Figure 5:**
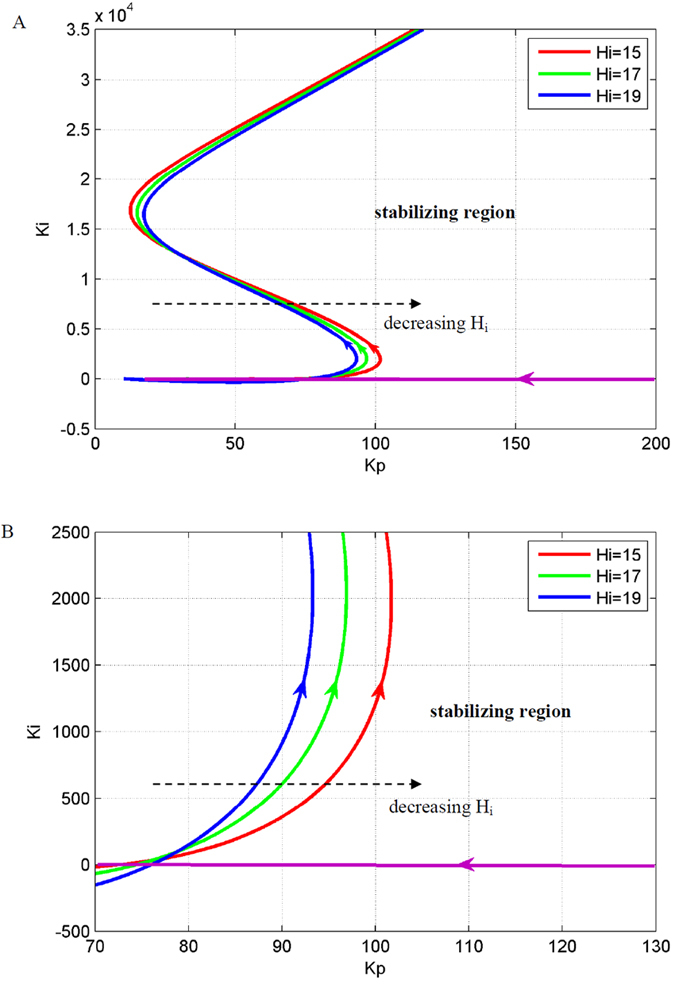
The effect of inhibitory parameter, *H*_*i*_, on the stabilizing region of the PI controller, (**B**) is an enlarged version of (**A**). The parameter space to the right of the red, green and blue curves are the stabilizing region of the PI controller for suppressing epileptic activity in the NMM with *H*_*i*_ = 15, 17 and 19, respectively.

Figures 4 and 5 illustrate the stabilizing regions of the NMM PI controller for different abnormal values of *H*_e_ and *Hi*, respectively. Figures 4B and 5B show the stabilizing region of the PI controller for small values of the integral coefficient, *K*_*i*_. The results demonstrate that the stabilizing region of the PI controller becomes smaller with increasing *H*_*e*_ (hyper-excitation) and decreasing *H*_*i*_ (low inhibition).

### Theoretical results of the steady-state performance analysis

According to Fig. 2C, we can derive the closed-loop transfer function of the PI-NMM from the input, *r*(*t*), to the error signal, *e*(*t*), as


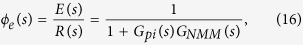


where *E*(*s*) and *R*(*s*) are the Laplace transforms of *e*(*t*) and *r*(*t*), respectively. According to Equation (16), one can obtain


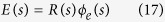


In this study, the desired output of the PI-NMM was set to a constant value, *r*(*t*) = *a*, and its Laplace transform is derived as follows:


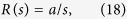


Substituting Equations (16) and (18) into Equation (17), we further obtain


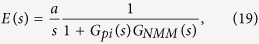


According to feedback control principles^47^, the control error at steady state, *e*_*ss*_, is as follows:


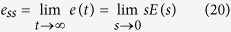


Substituting Equations (2), (4) and (19) into Equation (20), we derive


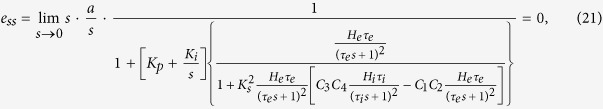


which shows that the output, *y*(*t*), of the NMM is equal to the desired output, *r*(*t*), at steady state.

**Figure 6:**
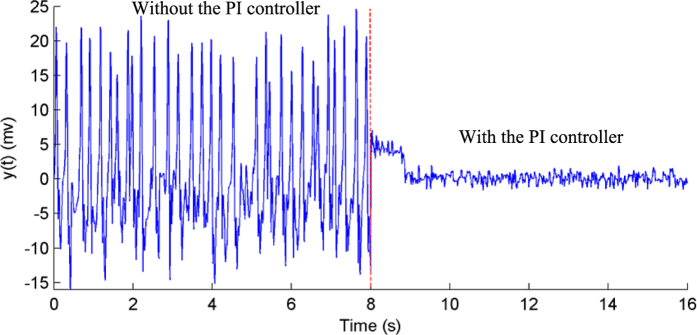
The output of the NMM without (before the eighth second) and with (after the eighth second) the PI controller during hyper-excitation (*H*_*e*_ = 7.0).

**Figure 7:**
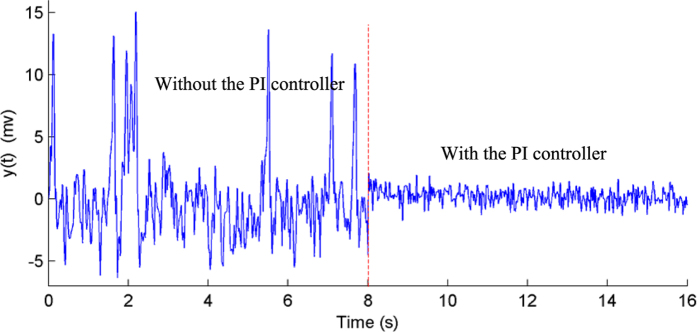
The output of the NMM without (before the eighth second) and with (after the eighth second) the PI controller during low inhibition (*H*_*i*_ = 17.0).

In this study, we set *r*(*t*) = *a* = 0; in this case, Equation (21) still holds. This result indicates that the PI controller can achieve an error-free control performance at steady-state and, thus, successfully suppresses epileptic activity in the NMM. It should be noted that the input of the NMM was noise signal; therefore, the PI-NMM control system could not be operated at steady-state^57^ and a small control error existed, as shown in Figs. 6 and 7.

### Simulation results

Simulations were conducted to illustrate the efficiency of the proposed PI control scheme to suppress epileptic activity in the NMM. We simulated the following two cases: hyper-excitation (*H*_*e*_ = 7.0 mV) and low inhibition (*H*_*i*_ = 17.0). According to Figs. 4 and 5, we determined the parameter values of the PI controller to be *K*_*p*_ = 310 and *K*_*i*_ = 2 for the hyper-excitation case and *K*_*p*_ = 90 and *K*_*i*_ = 2 for the low-inhibition case, respectively. The simulation results are illustrated in Figs. 6 and 7. The results showed that the output of the NMM without the PI controller was high-amplitude epileptic activity, which became low-amplitude activity under the control of the PI controller. Thus, the high-amplitude epileptic activities were successfully suppressed by the designed PI controller.

### Limitations

To determine the stabilizing region of the PI controller, we used the graphical stability analysis method and conducted a linearized approximation of the NMM sigmoid function, which is extensively used in previous NMM studies^27^,^46^,^58^,^59^,^60^. It should be noted that the linearized approximation may provide a conservative estimate of the PI controller stabilizing region. In the future, a non-linear method, such as the bifurcation analysis method^28^,^42^,^43^,^45^, should be employed to determine a more specific stabilizing region for the proposed PI-based control scheme.

## Conclusions

In the present study, we used Jansen’s neural mass model as a test bed to develop a systematic design approach to determine the control parameters of a PI controller. It should be noted that the proposed design method of the PI controller was independent of a specific model. Thus, it can be extended to other neural models^37^,^40^,^45^,^46^,^50^,^51^,^52^,^53^,^54^,^55^, such as the epileptic activity model developed by Wendling *et al*.^40^, and other low-order closed-loop controllers^24^,^25^,^26^,^27^,^28^,^29^,^30^,^31^, such as the proportional controller and the proportional-derivative controller. Here, we took a parametric approach to seizure behaviour to demonstrate how to use the PI controller to suppress epileptic activity. However, there are other factors that cause epilepsy^1^ in addition to the excitatory and inhibitory parameters, such as the stimulus^43^,^45^. The design method and process were not dependent on the factors that cause epilepsy; thus, the proposed design method can be extended to other cases involving epileptic activity that is characterized as high-amplitude oscillations^1^,^4^,^28^,^40^,^41^,^45^,^49^,^52^.

A graphical stability analysis method was utilized to determine the stability region of the PI controller in the parameter space. This provided a region of the PI control parameters that would suppress epileptic activity in the NMM. The proposed method ensured that the design of the controller was analytical, enabled theoretical analysis and revealed cause and effect relationships in a theoretical manner. This allowed us to explore the relationship between control parameters and model parameters that induced epileptic activity, which helped us understand the mechanism that suppresses neural diseases through closed-loop neurological electrical stimulation. In future work, we should attempt on-line seizure suppression by applying the proposed control scheme to standard animal models of epilepsy, with the long-term goal of applying it to human patients.

## Additional Information

**How to cite this article**: Wang, J. *et al*. Suppressing epileptic activity in a neural mass model using a closed-loop proportional-integral controller. *Sci. Rep.* **6**, 27344; doi: 10.1038/srep27344 (2016).

## Figures and Tables

**Table 1:** Physiological interpretation and standard values of the parameters in the NMM.

Parameter	Description	Standard value
*H*_e_	average excitatory synaptic gain	3.25 mV
*H*_i_	average inhibitory synaptic gain	22 mV
*τ*_*e*_	average synaptic time constant for excitatory subpopulation	0.0108 s
*τ*_*i*_	average synaptic time constant for inhibitory subpopulation	0.02 s
*C*_*1*_, *C*_*2*_	average number of synaptic contacts in the excitatory feedback loop	*C*_*1*_ = 135; *C*_*2*_ = 0.8 × 135
*C*_*3*_, *C*_*4*_	average number of synaptic contacts in the inhibitory feedback loop	*C*_*3*_ = 0.25 × 135*; C*_*4*_ = 0.25 × 135
*v*_0_*, e*_0_, r	parameters of nonlinear *S* function	*v*_0_ = 6 mV; *e*_0_ = 2.5 s ^−1^; r = 0.56 mV ^−1^
